# The effect of Ca and Bi addition on the mechanical strength, corrosion resistance, and Rechargeability of Pb1.5Sn alloy for the positive grid of lead-acid batteries

**DOI:** 10.1016/j.heliyon.2024.e38536

**Published:** 2024-09-26

**Authors:** Abdias Gomes dos Santos, Magda Rosângela Santos Vieira, Flávio José da Silva, Nadège Bouchonneau

**Affiliations:** Departamento de Engenharia Mecânica, Universidade Federal de Pernambuco – UFPE, CEP: 50670-901, Recife, PE, Brazil

**Keywords:** Lead-acid batteries, Positive grids, Alloy elements, Calcium and bismuth, Corrosion

## Abstract

Lead-acid batteries need to evolve to keep up with the electrification of vehicles and not lose ground to other technologies. The grid designed using a lead alloy thus plays a very important role in the performance of the battery, as, in the course of the various cycles, this component undergoes a natural corrosion process at positive potential, while immersed in a sulfuric acid solution. The aim of this study is therefore to examine the effect of the addition of calcium and bismuth on the microstructure, mechanical behavior and corrosion resistance of the Pb1.5%Sn alloy, with a view to using this in the positive grid of lead-acid batteries. The alloys developed during this study were evaluated using optical microscopy and scanning electron microscopy. Mechanical properties were investigated using universal tensile and hardness tests. Electrochemical tests of cyclic voltammetry, chronoamperometry and electrochemical impedance spectroscopy were carried out in a 5M sulfuric acid solution at 25 °C to simulate the behavior of the alloy when in operation. The composition of the corrosion products formed were subsequently characterized by morphological analysis using scanning electron microscopy and the composition was determined using energy dispersive X-ray spectroscopy and X-ray diffraction analysis. The results indicate that the Pb1.5Sn0.12Bi alloy presented better corrosion resistance characteristics than the Pb1.5Sn0.05Ca alloy, making it suitable for inclusion in the composition of the positive electrode of a lead-acid battery. Further investment is however required to compensate for the shortcomings in relation to the mechanical properties of the material.

## Highlights

• The Pb1.5Sn0.12Bi alloy exhibited higher corrosion resistance compared to the Pb1.5Sn0.05Ca alloy.
• The Bi and Ca elements contributed to the reduction of the alloy's grain size.
• Electrodes produced with Pb1.5Sn0.12Bi alloys showed corrosion products with higher conductivity.
• Over time, more conductive corrosion products could result in better performance from the battery's positive electrodes.

## 1 Introduction

Lead-acid batteries (LABs) are well-known on account of their extensive use in combustion engine vehicles. There is a worldwide supply chain of providers and manufacturers, and the recycling efficiency of such batteries is greater than 98 %, making them a sustainable and low-cost solution [1,2]. According to the International Lead Association (ILA), around 60 % of the LABs are used in the automotive sector. Stationary batteries are generally used for backup power supply like home appliances, data centers & telecommunication, forklifts, cranes, marines, etc. The global market for the LABs was 79.9 billion USD in 2021 and is forecasted to reach 115.1 billion USD during 2022–2030 with a compound annual growth rate of 2.52 %. The growth of LABs across the globe is expected to continue [[3], [4], [5], [6]].

Despite the advancement of modern systems like lithium-ion as well as nickel-metal hydride systems, lead-acid batteries are still the top choice for stationary applications, solar traffic lights, uninterruptible power supplies, hybrid electric vehicles and automobiles [1,7].

This technology, however, needs improvement, particularly in relation to recharging speed, increased cycle depth, and durability, so as to provide a larger number of ampere-hours in the course of a battery's lifespan, which, in combination with cost, could enable them to outperform other accumulator technologies, thereby ensuring longevity [1].

Lead-acid batteries provide several benefits, including affordability, good safety, minimal self-discharge, as well as manufacturing facilities [[8], [9], [10]]. The performance of battery grids is known to be significantly influenced by alloying content. Lead and lead alloy were generally utilized as framework materials in lead-acid batteries owing to their efficient anti-corrosion capabilities in H2SO4 medium. The use of pure Pb leads to the production of passive films of strong oxides at the interface between grid and energetic material [[11], [12], [13], [14], [15], [16]].

The heart of a lead-acid battery is its plates, which take the form of a grid, which conducts electrons from the active material during discharge or from an external source during charging, in a manner akin to the circulation of the blood in the human body, and provides sustainability for the active material, in a manner analogous to the backbone, and the active material itself, in which electrochemical reactions occur to produce electricity [17,18].

The design of the grid must take into consideration, not only these dimensional parameters, but also the choice of material, which is crucial for the performance of the battery, since it will naturally corrode over time and the reversibility of processes that take place during charge/discharge determines the cycle life of batteries [18]. The knowledge of the mechanical and electrochemical properties of the material helps the designer to select the best material for a given application [19,20].

During the evolution of lead batteries, calcium and tin alloys were introduced, ushering in an era of maintenance-free (water replacement) batteries. Calcium made it possible to achieve mechanical strength by replacing antimony alloys. Alloys containing calcium are nowadays the main component used in the battery industry [21]. Bismuth has, however, been suggested as an alternative for the creation of alloys for grids with increased corrosion-resistance, thereby allowing for greater component longevity [[22], [23], [24], [25]].

The key innovation of this work resides in the use of a Pb1.5%Sn base alloy and the integration of both mechanical and electrochemical approaches. Unlike existing studies on PbBi alloys, which do not provide mechanical information, and research on PbCa alloys, which typically focus solely on mechanical properties, this study also investigates the electrochemical aspects and corrosion products. The present study aims to characterize Pb1.5Sn0.05Ca and Pb1.5Sn0.12Bi alloys. The formation of the alloy was accomplished by using the reference alloy commonly applied in the battery industry, containing 1.5 % Sn and 0.05 % Ca, and with the calcium replaced by 0.12 % Bi, a value of bismuth corresponding to double the amount needed to observe the effects of bismuth in a lead alloy, according to the study conducted by Rice [24].

## 2 Materials and methods

### 2.1 Preparation of alloys and Fabrication of specimens

The alloys were prepared by way of a casting process, using high-purity metals: lead, tin, calcium, and bismuth, all with purity greater than 99.99 %. A crucible with a capacity of 75 kg was heated by electric resistance and temperature was controlled using thermocouples (Type J). The alloying elements—tin (Sn), calcium (Ca) and bismuth (Bi)—were added to the lead. A mechanical stirrer was used to homogenize the molten mass. Specimens were produced in the form of 10 mm-thick circular discs with a diameter of 30 mm and were prepared in accordance with the ASTM E8 standard [10]. The composition of the alloys used was validated using an optical emission spectrometer (SPARK). In this study, the alloy was produced by using the reference alloy commonly applied in the battery industry with 1.5%Sn and 0.05%Ca, and with the calcium being replaced by 0.12%Bi. This value was chosen in order to double the amount needed to observe the effects of bismuth in a lead alloy, as highlighted by Rice [24]. Table 1 shows the average obtained from three consecutive analyses using the SPARK method, thus ensuring the correct composition of the sample under study.

**Table 1:** Analysis of Composition of Alloys Prepared using the SPARK Method.

Alloy	% of Elements			
	Pb	Bi	Sn	Ca
**Pb1.5Sn0.05Ca**	98.45	0	1.5	0.05
**Pb1.5Sn0.12Bi**	98.38	0.12	1.5	0

### 2.2 Metallographic analysis

Samples were embedded in Araldite epoxy resin and ground using SiC metallographic paper at consecutively larger granular sizes of 320, 600, and 1200#. Diamond paste polishing was subsequently applied, at granular sizes of 3 μm and 1 μm respectively. The specimens were washed with water and dried at room temperature. The surfaces were etched with Pollack reagent (citric acid, ammonium molybdate, and water) by dripping, without heating, for 30s [21]. The specimens were then washed with water, followed by ethanol, and finally dried in a jet of hot air. Images were captured using an Olympus microscope, model BX 51, with a camera attached, and the Olympus Stream software package.

### 2.3 Tensile test

Tensile tests were performed using an EMIC universal tensile testing machine with a maximum capacity of 100 kN. A GR001 type grip with a maximum capacity of 5 kN was also used, along with a 5-kN-load cell model CCE5KN and an EE04 10671 type EE04 10671 differential electronic extensometer. Test displacement was set at 2 mm/mm up to the point where the specimen fractured. The results obtained were treated in accordance with ASTM International's Standard Test Methods for Tension Testing of Metallic Materials, ASTM E8-04 [25].

### 2.4 Microhardness test

Microhardness analyses were conducted using an EMCOTEST DuranScan 10G5 model microhardness tester, with a square-based pyramidal indenter with 136° between faces. A 10g load was applied to the polished surface, and six indentations were made in different regions of the sample. The average reading obtained was adopted as the result. The results obtained were subsequently treated in accordance with ASTM International's Standard Test Methods for Rockwell Hardness of Metallic Materials, ASTM E18-22 [26].

### 2.5 Electrochemical tests

All electrochemical tests were conducted in triplicate, using Avesta corrosion cells manufactured by Biologic, containing 500 mL of sulfuric acid at a concentration of 5 M, at an average temperature of 25 °C. A BioLogic SAS VSP 1246 model potentiostat with an EC-LAB 11.01 interface was used for parameter control and data acquisition. The corrosion cell employed consisted of three electrodes: a working electrode with an area of 1 cm^2^, corresponding to the materials under investigation (Pb1.5Sn0.05Ca and Pb.5Sn0.12Bi), a graphite rod as the counter electrode, and a Saturated Calomel Electrode (SCE) as the reference electrode.

The working electrodes were washed three times with deionized water and ethanol to remove any dirt or grease and then dried. They were subsequently weighed on an AUY220 model Shimatzu analytical balance with a sensitivity of 0.1 mg. The specimens were then mounted in the corrosion cell.

Cyclic voltammetry is the technique most used by researchers to simulate a positive electrode in operation and thus to predict behaviors during the use [[27], [28], [29], [30], [31], [32]]. Prior to electrochemical tests, the working electrode was kept at a potential of −1.3 V for 10 min to remove the oxides that had formed during the preliminary surface treatment. The material subsequently underwent cyclic voltammetry testing, at potentials ranging from +1.3 V to +2.2 V (vs SCE), at a scan rate of 10 mV/s, giving a total of 400 cycles evaluating the electrochemical activity of the corrosion layer formed on the materials studied.

After cycling testing, electrochemical impedance spectroscopy (EIS) analysis was performed at a potential of +2.2 V (vs SCE), with a frequency scan from 100 kHz to 100 mHz and an amplitude of 10 mV.

After the EIS test for cycle 400, the alloys were pickled in a solution of 80 g sodium hydroxide, 18 g mannitol, 8 g hydrazine dichloride, and 800 mL deionized water. After pickling, the samples were cleaned and re-weighed to evaluate the loss of mass resulting from the corrosive process.

The morphology and composition of the deposits formed during the cycling process were assessed using chronoamperometry tests to obtain corrosion products at potentials of +1.3 V, +1.7 V, and +2.2 V (vs SCE). Different samples were subjected to each of these potentials for a period of 6 h for subsequent scanning electron microscopy (SEM) and X-ray diffraction (XRD) analysis.

### 2.6 X-ray diffraction analysis and energy dispersive X-ray spectroscopy

The crystalline phases of the corrosion product deposits generated in the electrochemical tests were identified using XRD analyses at potentials of +1.3 V, +1.7 V, and +2.2 V (vs SCE) following chronoamperometry. A 3-kW Shimadzu XRD-7000 diffractometer using CuKα radiation with a voltage of 40 kV and a current of 30 mA was employed. The step applied was 2θ/second, with the angle ranging from 5° to 80°.

Complementing the results obtained from X-ray Diffraction (XRD), Energy Dispersive X-ray Spectroscopy (EDS) was performed with the aim of identifying each element present and quantifying the elemental composition of the corrosion products of the studied alloys.

### 2.7 Scanning electron microscopy

The corrosion mechanism was evaluated using a Hitachi TM-3000 model benchtop SEM to examine the surface of the samples once these had been pickled to remove the corrosion layer. The morphology of the corrosion products deposited on specimens undergoing chronoamperometry was meanwhile assessed using an SEM with a field emission gun (FEG) as the emission source.

## 3 Results and Discussion

### 3.1 Metallographic analysis

**Fig. 1:**
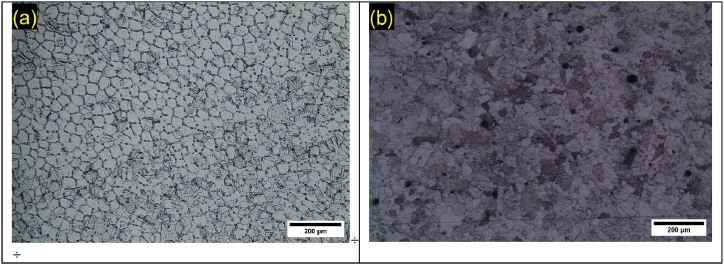
Metallographic result for Pb1.5Sn0.05Ca and Pb1.5Sn0.12Bi alloy, according to International's Standard Test Methods for Determining average Grain Size, ASTM E112-24 [33].

Fig. 1 presents the microstructures obtained by optical microscopy for Pb1.5Sn0.05Ca and Pb1.5Sn0.12Bi alloys. The procedure to estimate the average grain area was the International's Standard Test Methods for Determining Average Grain Size, ASTM E112-24 [33]. The addition of calcium to the alloy causes the formation of a microstructure resembling an acicular structure, a solid solution with a lead-rich matrix and alloy-rich boundaries, as shown in Fig. 1a. According to Osório et al. [19], alloys composed of PbSn alone have coarse grains, indicating the grain-refining effect of calcium.

The addition of bismuth produces a grain-refining effect of lower intensity compared to calcium, producing the acicular morphology shown in Fig. 1b. According to Rice [24], the addition of 0.1%Bi to lead as an alloying element has a grain-refining effect. The same result was obtained in the present study, where the grain size was reduced when bismuth was added, compared to the PbSn alloy studies presented by Osório et al. [27].

### 3.2 Tensile test

**Fig. 2:**
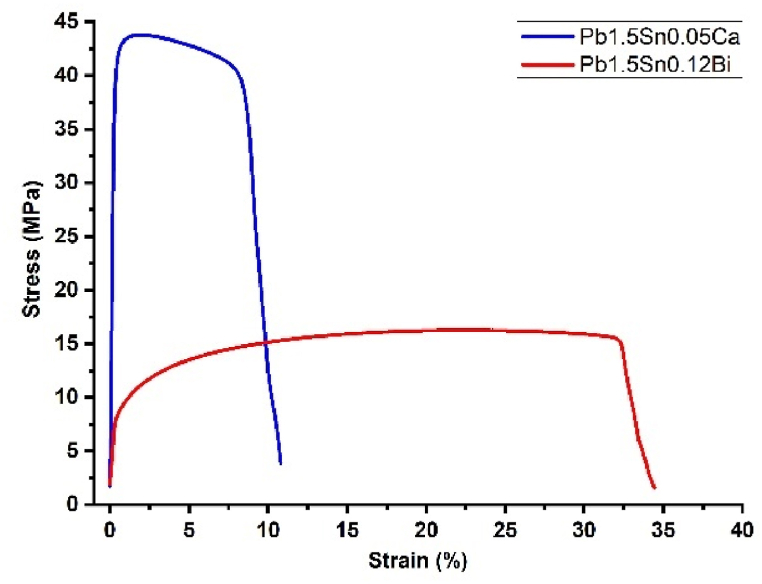
Result of tensile test of Pb1.5Sn0.05Ca and Pb1.5Sn0.12Bi alloys according to the test ASTM E8-04 [25].

During the production of battery electrodes, alloys can be melted, shaped, stamped, rolled, or drawn out according to the specific method employed to manufacture the grid [20]. The tensile test is able to predict certain processability behaviors of the alloys during the manufacturing process. Fig. 2 illustrates a stress-strain curve for each alloy investigated, and below are presented the results of the average of 3 samples and the respective standard deviation obtained in the tensile test as reported by the ASTM International's Standard Test Methods for Tension Testing of Metallic Materials, ASTM E8-04 [25].

The presence of calcium in PbSn alloys enables the development of the Pb_3_Ca and Sn_3_Ca phases. Depending on the proportion of calcium and tin, an organization described as 'puzzling' is established in the alloy. This impedes dislocations and consequently gives rise to a significant increase in the mechanical resistance of the material, as reflected in yield strength. It also causes an increased Young's modulus and ultimate tensile strength, and a gradual decrease in deformation up to the point at which the material fails [18,21].

According to studies of PbSn alloys conducted by Rice and Chen et al. [24,28], the addition of bismuth does not provide the alloy with additional mechanical properties to any significant degree. The values observed were much lower than those obtained in the presence of calcium. The figures reported in the present study are consistent with those presented in the literature.

The results show that the alloy containing bismuth exhibits greater ductility compared to a Pb1.5Sn0.05Ca alloy. Therefore, it is necessary to use machinery that applies lower stresses to the material during operation, compared to machinery designed for processing calcium-containing alloys, in order to ensure the geometric integrity of the product.

### 3.3 Microhardness analysis

**Fig. 3:**
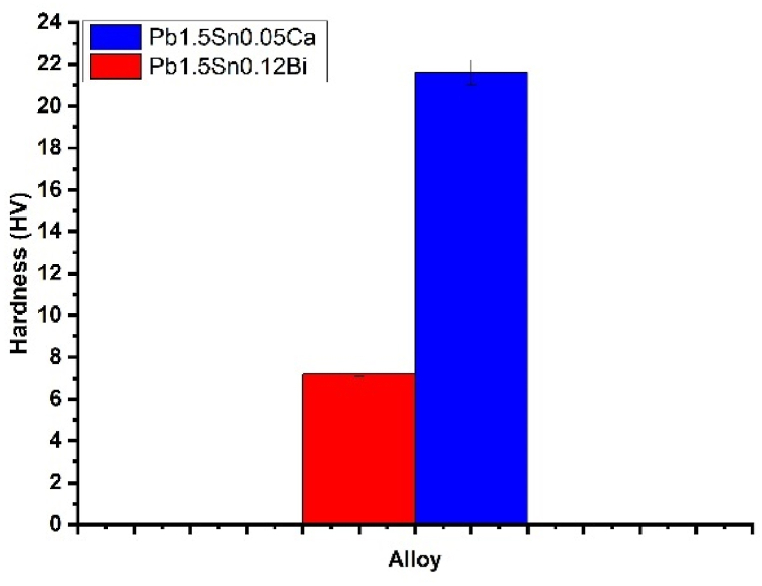
Hardness of Pb1.5Sn0.05Ca and Pb1.5Sn0.12Bi alloys.

The production of electrodes for batteries involves the grids that will be used as electrodes being constantly moved under racks and carried on conveyor belts. This may lead to material wear and tear [20,34]. Vickers microhardness tests serve as a reference for predicting the surface wear of a material and these were conducted as outlined in the ASTM International's Standard Test Methods for Rockwell Hardness of Metallic Materials, ASTM E18-22 [26]. The results are presented in Fig. 3.

The calcium alloy presented significantly greater hardness than the alloy containing bismuth. The results corroborate those provided by the tensile test and indicate that the addition of calcium improves the mechanical behavior of the alloy.

### 3.4 Cyclic voltammetry analysis

**Fig. 4:**
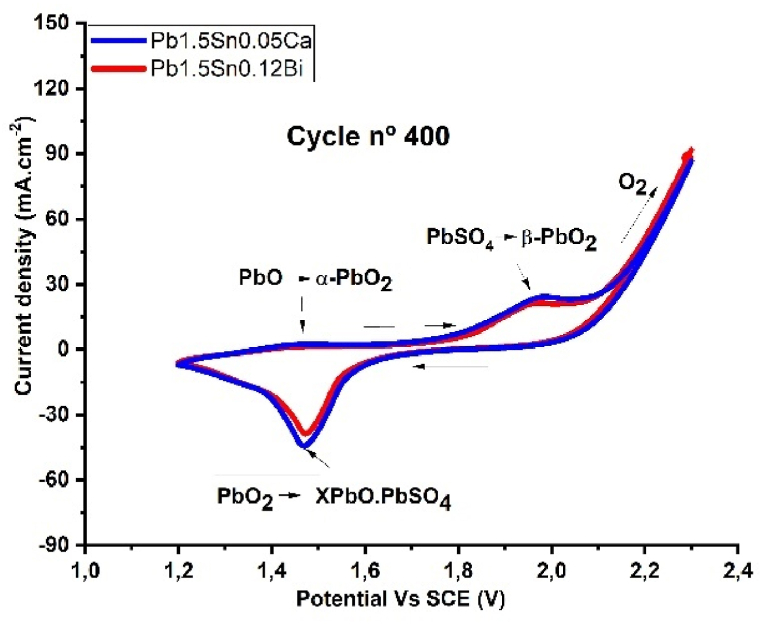
Cyclovoltammograms for Pb1.5Sn0.05Ca and Pb1.5Sn0.12Bi alloys at potentials ranging from 1.3 to 2.2 V (vs SCE), in a solution of H_2_SO_4_ (5 M). Cycle number: 400. Scan rate: 10 mV s^−1^.

During operation in an electrical accumulator, anodic potential shifts to more positive values during the charging process and to more negative ones during battery discharge. The grid materials are exposed to an electrolyte composed of sulfuric acid diluted to approximately 5M, with the potential of these electrodes varying between +1.2 and + 2.2V (vs SCE) during cycling. During the cycles, the base material undergoes oxidation, leading to the formation of a corrosion layer. The alloys Pb1.5Sn0.05Ca and Pb1.5Sn0.12Bi were simulated. Fig. 4 presents the voltammogram according to ASTM G59-97 [35], obtained in the 400th cycle, where the corrosion layer has formed, for the study of the electrochemical phenomena of the alloys under investigation.

Fig. 4 shows the voltammograms obtained for Pb1.5Sn0.05Ca and Pb1.5Sn0.12Bi at cycle 400.

The voltammogram in the anodic polarization region (upper region) shows the conversion of PbSO_4_ and PbO crystals into active PbO_2_ material. This phenomenon occurs during the battery charging phase. In the presence of sulfuric acid solution, the Pb1.5Sn0.05Ca and Pb1.5Sn0.12Bi alloys experience sulfation, resulting in the formation of a layer of PbSO_4_ crystals that impedes the passage of sulfate ions. After the formation of this layer, the pH inside the pores increases from neutral to alkaline, enabling a basic formation, with lead oxide in the base material. The increase in quantities of lead oxide displaces the sulfate crystals, allowing the passage of sulfate ions and restarting the process of layer growth of PbSO_4_, PbO, and their compounds. The phenomenon of local pH increase in the positive electrodes of lead batteries has been discussed extensively by Yang and Pavlov [17,35].

During anodic displacement, the Pb1.5Sn0.05Ca and Pb1.5Sn0.12Bi alloys first convert the material inside the pores in the region of lower pH, where the formation of α-PbO_2_ is favored, as indicated by the peak obtained at a potential close to 1.5V. The second anodic peak occurs around 1.95V and corresponds to the oxidation of PbSO_4_ in more acidic regions, where the formation of β-PbO_2_ is favored. This conversion phenomenon has been observed in various studies [17,[36], [37]].

At higher potentials during the oxidation of the PbSn alloy, evolution of gaseous oxygen on the surface of the working electrode can be observed, as shown in the reactions described, respectively, by Equations (1), (2)).

(1) $$2H_{2}O\left(l\right)↔\;O_{2}\left(g\right)+4H^{+}\;\left(aq\right)+4e^{-}$$

(2) $${2H}^{+}\left(\text{aq}\right)+2e^{-}\;↔\;H_{2}\left(g\right)$$

The evolution of oxygen occurring during these reactions has a deleterious effect on the operation of the battery. O_2_ favors the formation of PbO, which is a material of low conductivity, thus hindering the conversion of PbSO_4_ into PbO_2_. This phenomenon creates accumulator charge acceptance deficiency. In addition to this, hydrolysis results in further concentration of the solution, which comprises dilute acid. As water is lost, the concentration of acid in the solution increases, thereby hindering the recharging of the battery, owing to intensification of the sulfation process [[17], [35], [38], [39], [40]].

During accumulator discharge, the potential of the electrode shifts to lower polarization. In the cathodic direction (in the lower region), a single peak indicative of PbO_2_ reduction to lead sulfates of the XPbO.PbSO_4_ type could be observed. Similar results to those of the voltammograms obtained for Pb1.5Sn0.05Ca and Pb1.5Sn0.12Bi alloys can be found in the literature on lead and its alloys [[41], [42], [43], [44], [45], [46]].

In both alloys, the second anodic peak was found to be larger than the first. This phenomenon can be attributed to the layer of PbO formed in the inner region being smaller than the layer of PbSO_4_ formed in the outer region of the electrode. The β-PbO_2_ formation peak was more pronounced in the Pb1.5Sn0.05Ca alloy than in Pb1.5Sn0.12Bi, indicating greater electroactivity in the conversion of PbSO_4_ to PbO_2_.

The cathodic peak, indicating the conversion of PbO_2_ toXPbO.PbSO_4_ compounds, confirms the presence of higher electrochemical activity in the Pb1.5Sn0.0.5Ca alloy, showing that calcium has greater corrosive effect than bismuth in the Pb1.5Sn0.12Bi alloy. The increased oxygen evolution in the bismuth-containing alloy does not correspond to greater corrosion of the alloy, even though it serves as a consumable in the oxidation reaction of the material. It is possible to observe from the cathodic peak that the electrochemical activity of the conversion of PbO2 to XPbO.PbSO_4_ was lower.

The associated problem concerns the loss of water present in the battery electrolyte, which accelerates the failure mode compared to the calcium-containing alloy. This issue can be addressed by adding water to the battery in the case of maintenance-free batteries, or by adding additives to the electrolyte that can reduce water consumption to acceptable levels, making the Pb1.5Sn0.12Bi alloy suitable for use.

### 3.5 Evaluation of the corrosion mechanism

The extent of loss of mass in Pb1.5Sn0.05Ca and Pb1.5Sn0.12Bi samples after cyclic voltammetry (cycle 400) was evaluated. A further study was conducted to confirm the existence of a more extensive corrosion layer in the Pb1.5Sn0.05Ca alloy following the cycles. The alloy containing calcium exhibited a loss of mass of (14.8 ± 0.7 mg), this being greater than that observed in the alloy containing bismuth (12.9 ± 1.0 mg). This result indicates more extensive corrosion of the Ca-containing alloy, resulting from more extensive formation of corrosion product deposits on the surface of the material. These findings corroborate the peaks obtained using voltammetry. Analysis of the corrosion mechanisms at work in the samples may help to shed some light on these results.

**Fig. 5:**
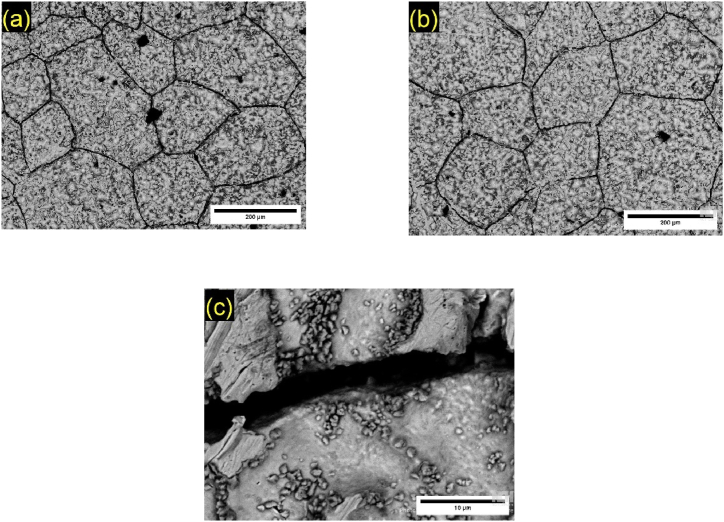
SEM of Pb1.5Sn0.05Ca alloy (a) and Pb1.5Sn0.12Bi alloy (b) after removal of the corrosion layer and detail of intergranular corrosion (c).

Micrographs of the test specimens obtained using SEM at the end of the cyclic voltammetry test and subsequent acid etching can be seen in Fig. 5. Analysis of alloys revealed that corrosion tended to occur mostly in the intergranular regions, corroborating the findings of two studies conducted by Burashnikova and colleagues [29,30].

### 3.6 Morphological analysis of corrosion deposits using SEM

**Fig. 6:**
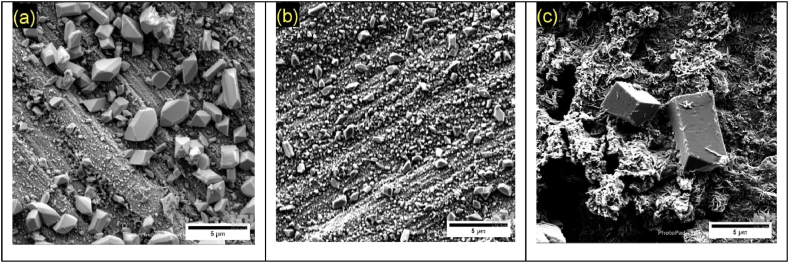
Morphology of corrosion deposits of Pb1.5Sn0.05Ca, after chronoamperometry tests at electrochemical potentials of (a) +1.3 V; (b) +1.7 V, and (c) +2.2 V (vs ECS).

**Fig. 7:**
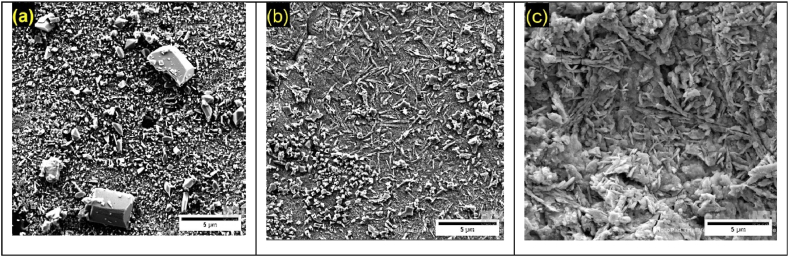
Morphology of corrosion deposits of Pb1.5Sn0.12Bi, after chronoamperometry tests at electrochemical potentials of (a) +1.3 V; (b) +1.7 V; and (c) +2.2 V (vs ECS).

SEM analyses of the surfaces after chronoamperometry were conducted to assess the morphology of corrosion deposits formed at different potentials. Fig. 6, Fig. 7 show, respectively, the micrographs for Pb1.5Sn0.05Ca and Pb1.5Sn0.12Bi alloys at electrochemical potentials of +1.3 V, +1.7 V, and +2.2 V (vs ECS).

The surface of Pb1.5Sn0.05Ca showed PbSO_4_ crystals at potentials of +1.3 V and +1.7 V. These morphologies are similar to those found by Yangl. and Burashnikova et al. [17,30].

Analysis of the images in Fig. 6 (a) and (b) show the heterogeneity of the size of the crystals formed at a potential of +1.3 V, revealing large crystals and a layer of small crystals on the surface of the material. At a potential of +1.7 V, the size of the sulfate crystals is smaller, and this may be attributable to the conversion of part of the material into α-PbO_2_, thereby improving the conductivity of the material. According to studies carried out by Rice [11], the PbSn alloy also exhibited reduced crystal sizes when the potential increased from +1.3 V to +1.7 V. This phenomenon may also be explained by the formation of SnO, which is a semiconductor and thus improves electrical mobility in the newly formed layer.

Underneath this layer of sulfate crystals, PbO deposits and other basic Pb compounds, created by the reaction of PbO and PbSO_4_ may also be formed [17].

Fig. 6c shows that, at a potential of +2.2 V, most of the sulfate crystals were found to have dissolved. Two large crystals were, however, also observed. This shows that the formation of large crystals associated with a restricted area of contact with the PbO_2_ layer may result in incomplete conversion of these crystals, since a large amount of energy is required for such conversion. This result would, in practice, result in a decrease in the efficiency of the electrical accumulator, since sulfate crystals have low conductivity and occupy a more extensive portion of the electrode surface than PbO_2_ [34]. The PbO_2_ layer exhibited a porous morphology with cavities caused by the oxygen evolution process.

Fig. 7 shows the various different morphologies of Pb1.5Sn0.12Bi alloys. At a potential of +1.3 V, sulfate crystals can be seen to be present, and the number of large crystals formed is smaller compared to the Pb1.5Sn0.05Ca alloy.

At a potential of +1.7 V, a reduction in the number of PbSO_4_ crystals can be observed, with effects similar to those occurring with the Pb1.5Sn0.05Ca alloy, resulting in increased conductivity of the lower PbO layer. In addition to the PbSO_4_ crystals located in the lower left-hand corner of the image, an acicular morphology caused by lead carbonates located in the central region is also present. This material has not been so frequently reported in the literature, and its formation can be accounted for, primarily, by α-PbO_2_ and β-PbO_2_ compounds coming into contact with CO_2_ in the air. It can thus be inferred that the aforementioned phases were present prior to the formation of basic lead carbonate.

At potentials of up to +2.2 V, a more compact layer of PbO_2_ is observed. This morphology can be explained by the reduced formation of layers of PbSO_4_ giving rise to the rapid creation of PbO_2_ from the material. Lead carbonates are again found to be present. This can be explained by the fact that, at a potential of +2.2 V, the electroactivity of the surface of the material surface is even higher than at +1.7 V.

### 3.7 Compositional analysis of corrosion deposits by energy dispersive X-ray spectroscopy and X-ray diffraction

**Fig. 8:**
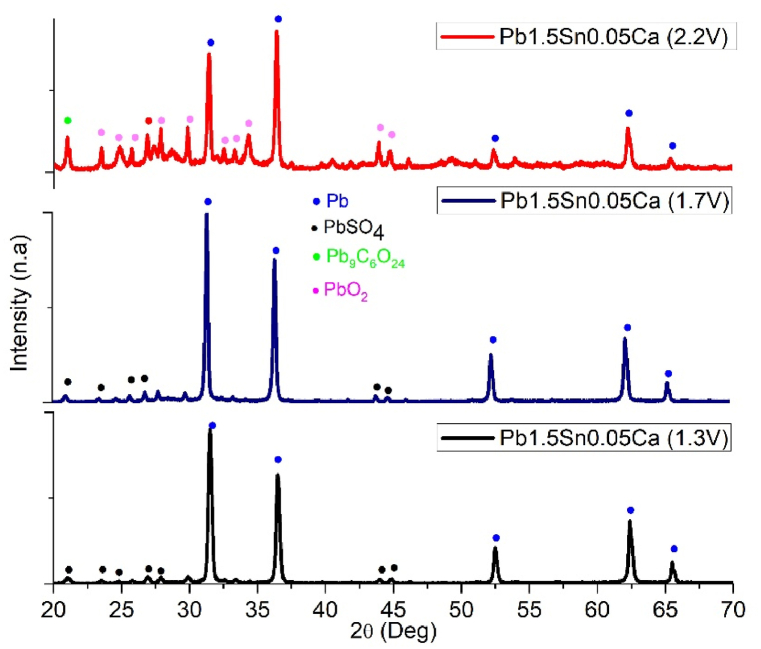
X-ray diffractograms for the Pb1.5Sn0.05Ca alloy at different chronoamperometry steps: +1.3V; +1.7V; and +2.2V (vs ECS), JCPDS reference number to (Pb, 96-101-1120), (PbSO4, 96-101-0951), (Pb9C8O24, 96-154-2100) and (PbO2, 96-900-5762) [47].

**Fig. 9:**
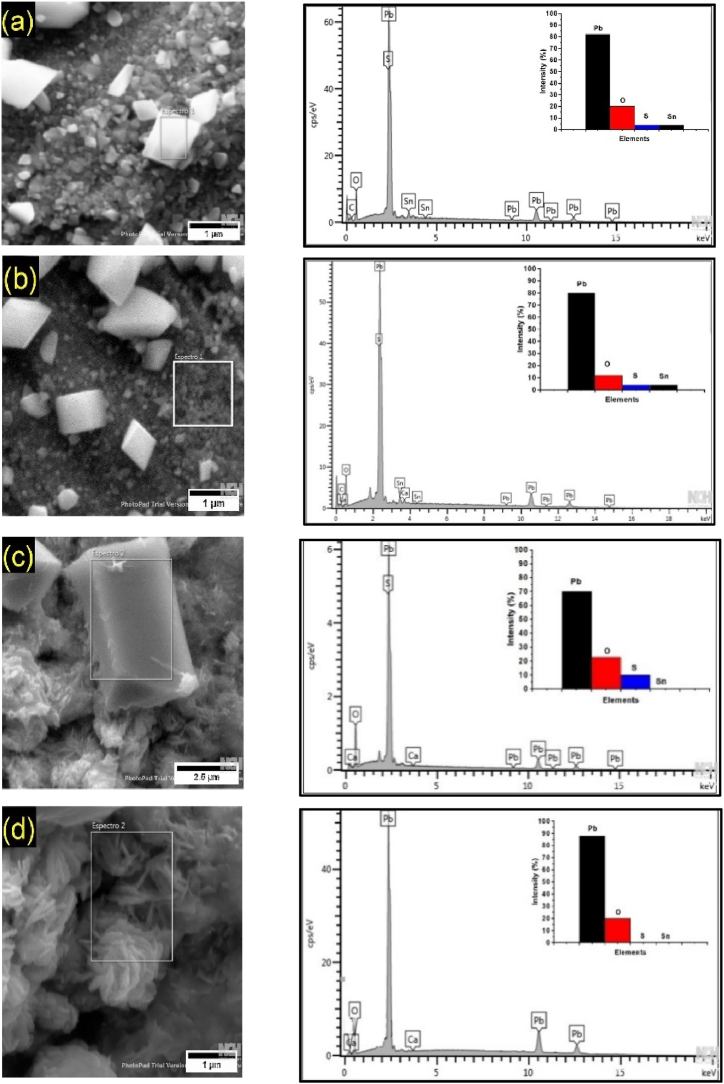
EDS analysis of the main components of the obtained morphologies in Pb1.5Sn0.05Ca alloy. (a)crystal with large size; (b)crystal with small size obtained in +1,3 and + 1,7V; (c)crystal with large size and (d)material formed at +2,2V (vs ESC).

Analysis of the corrosion products obtained with different alloys was carried out using X-ray diffraction (XRD), with the test specimens exposed to chronoamperometric steps at potentials of +1.3, +1.7, and +2.2V (vs ECS). Fig. 8, Fig. 9 show the diffractograms and analysis of corrosion deposits analysis obtained for Pb1.5Sn0.05Ca alloy.

XRD analysis of Pb1.5Sn0.05Ca identified the presence of PbSO_4_ at potentials of +1.3V and +1.7V (vs ECS). Peaks corresponding to PbSO_4_ could not be observed at a potential of +2.2V, while peaks associated with PbO_2_ and lead carbonate were visible.

At potentials below +1.3V, the predominant product was PbSO_4_. This product is formed by dissolution-precipitation, whereby metallic Pb is oxidized to Pb^2+^ ions that diffuse through the solution. Combining with SO_4_^2−^ ions, they reach a critical saturation point, precipitating on the surface of the alloy in the form of PbSO_4_ crystals. Below the surface, oxidation of Pb to Pb^2+^ occurs through the existing pores.

Oxidation of Pb to Pb2+ continues, as electrolyte (H_2_SO_4_) contact with Pb is still possible through existing pores, despite such access becoming more restricted [17,22]. The oxygen evolution process causes gas to diffuse through the pores in the direction of the substrate, bringing about a localized increase in pH and providing a conducive environment for subsequent formation of alkaline compounds such as PbO and basic lead sulfates. Growth of PbSO_4_ crystals and deposition of PbO within the pores will, therefore, simultaneously occur, and this will, through oxidation of Pb2+ to Pb4+, respectively, give rise to α-PbO2 and β-PbO2 [17,34].

At a higher potential (+1.7V), the conversion of the Pb1.5Sn0.05Ca alloy to PbSO_4_ and PbO intensifies. The formation of α-PbO2, resulting from the conversion of PbO beneath the sulfate layer was expected to occur at a potential of +1.7V. PbO and α-PbO2 were not detected at potentials +1.3V and +1.7V, respectively and their absence can be attributed to the small amount of PbO formed during the 6-h chronoamperometry test. Similar results were observed in PbSn alloys in the studies carried out by Santos et al. [21] and can be explained by the formation of small amounts of SnO competing with the formation of PbO. At +2.2V, the peaks characteristic of PbSO_4_ are no longer visible, indicating conversion of PbSO_4_ to β-PbO_2_. The absence of PbSO_4_ at this potential, despite being observed using SEM, can be attributed to the small quantities present.

Energy Dispersive X-ray Spectroscopy (EDS) analysis of the corrosion products of the Pb1.5Sn0.05Ca alloy was conducted to assess the elemental composition, corroborating the results from X-ray Diffraction (XRD). The results are presented in Fig. 9.

The EDS results show that at potentials of +1.3V and +1.7V, the formed crystals are primarily composed of Pb, O, and S (Fig. 9a–b). The crystal found at a potential of +2.2V has the same composition. It is a crystal that has not been converted (Fig. 9c), and the corrosion product material is composed of Pb and O (Fig. 9d). The results corroborate the findings obtained from X-ray Diffraction (XRD) and are consistent with other materials reported in the literature [17,34].

**Fig. 10:**
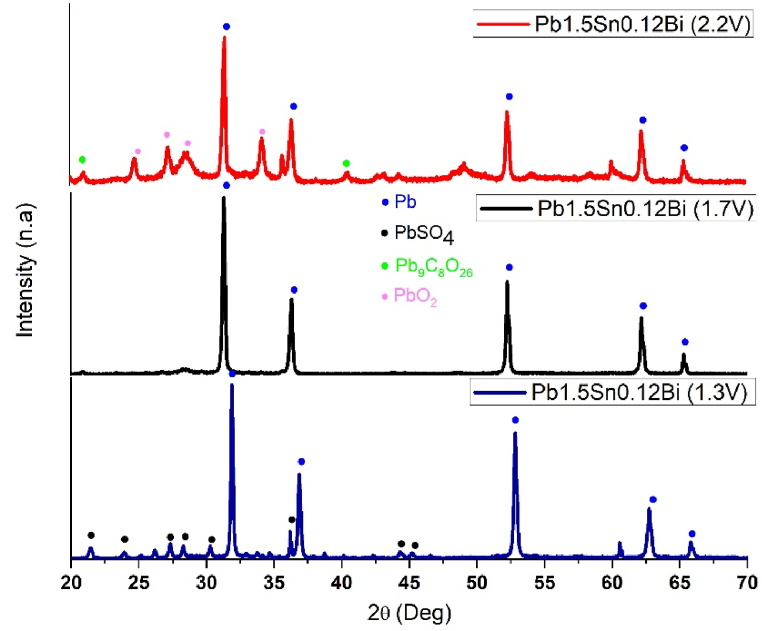
X-ray diffractograms for the Pb1.5Sn0.12Bi alloy at different chronoamperometric steps: +1.3 V; +1.7 V; and +2.2 V (vs ECS), JCPDS reference number to (Pb, 96-101-1120), (PbSO4, 96-101-0951), (Pb9C8O24, 96-154-2100) and (PbO2, 96-900-5762).

**Fig. 11:**
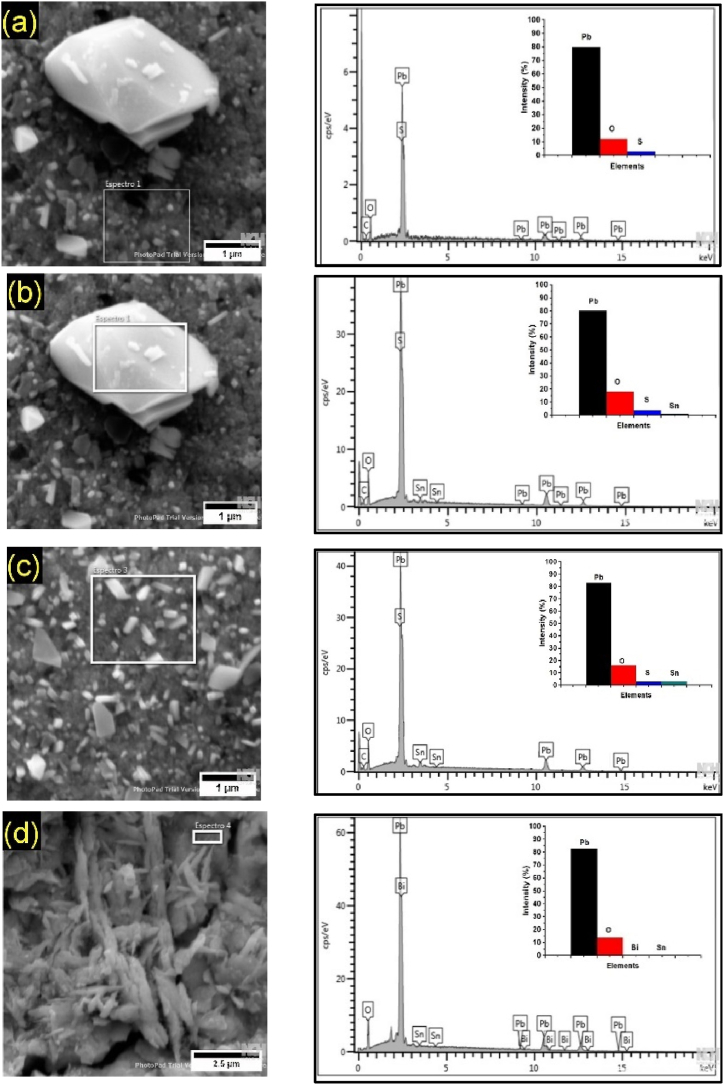
EDS analysis of the main components of the obtained morphologies in Pb1.5SnSn0.12Bi alloy. (a)cristal with small size; (b)cristal with large size obtained in +1,3; (c)cristal with small size obtained in +1,7 (d)material formed at +2,2V (vs ESC).

Fig. 10, Fig. 11 show the diffractograms and analysis of corrosion deposits analysis obtained for Pb1.5Sn0.12Bi alloy, the peaks characteristic of PbSO_4_ identified at a potential of +1.3V in the Pb1.5Sn0.12Ca alloy, as also seen in Pb1.5Sn0.05Bi.

According to the SEM data, the presence of bismuth inhibits the formation of sulfate crystals, in a manner consistent with the findings of Rice [24]. With no layer of sulfate crystals, production of the PbO layer is hindered under the conditions required for its growth. At a potential of +1.3V, only PbSO_4_ is observed.

At a potential of +1.7V, the difficulty of forming PbSO_4_ and PbO layers makes it impossible to identify a significant amount of any compound for XRD analysis. This indicates that the sulfate crystals underwent reduction, as is expected for PbSn alloys, and underlines the resistance to sulfation of the material surface conferred by bismuth. This means that bismuth element is a potentially effective additive for use in electrical accumulators. PbO2 was identified at a potential of +2.2V. High potential causes oxidation of the base material, explaining the compact morphology observed under SEM. No PbSO_4_ or PbO crystals were observed. Peaks indicating the formation of lead carbonate were identified where PbO_2_ had previously been present. The explanation for this has already been discussed in the results of the SEM analysis.

Similarly to the analysis performed for the Pb1.5Sn0.12Ca alloy, elemental analysis of the corrosion products formed in the Pb1.5Sn0.12Bi alloy was conducted to assess the elemental composition and complement the results obtained from X-ray Diffraction (XRD) analysis. The results are presented in Fig. 11.

The EDS results for the Pb1.5Sn0.12Bi alloy showed convergence with the results obtained from X-ray Diffraction (XRD). At potentials of +1.3V and +1.7V, both small (Fig. 11a and c) and large (Fig. 11b) crystals are primarily composed of Pb, O, and S. At a potential of +2.2V, the material is composed of Pb and O (Fig. 11d). The results were similar to those obtained with the Pb1.5Sn0.05Ca alloy. Other authors in studies of lead alloys also reported the predominance of the same corrosion products as those presented [17,23].

### 3.8 Impedance spectroscopy

**Fig. 12:**
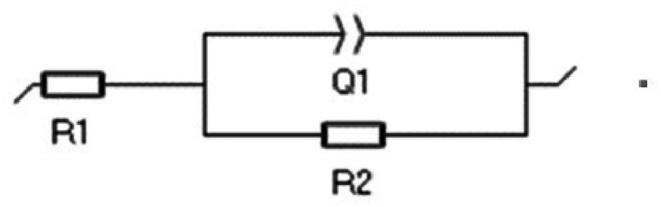
Equivalent circuit for explaining EIS phenomenon.

Based on previous studies [48,49], an equivalent circuit as presented in Fig. 12 was proposed as a way of shedding further light on the phenomenon.

**Fig. 13:**
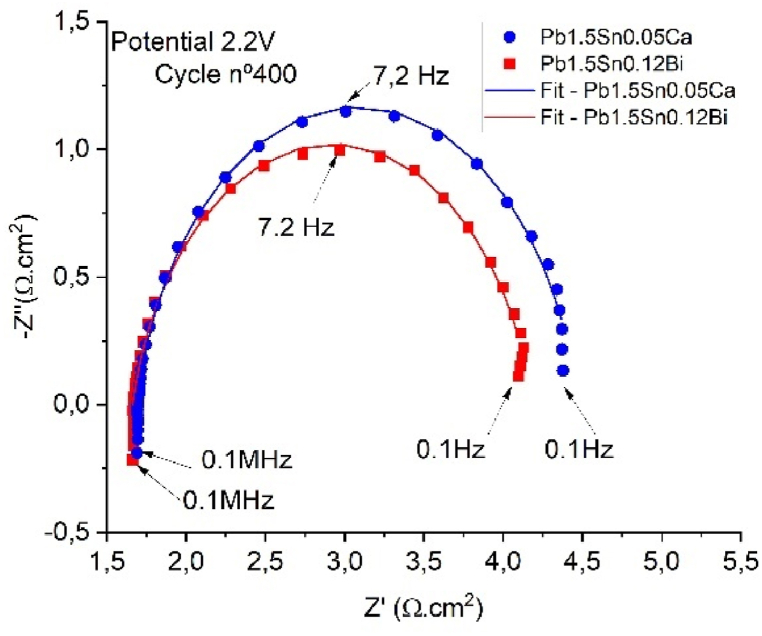
Nyquist plots for Pb1.5Sn0.05Ca and Pb1.5Sn0.12Bi alloys with corrosion deposit films formed after cyclic voltammetry test (cycle 400).

R1 represents the resistance of the electrolyte, Q1 represents the constant phase element (CPE), referring to the double corrosion layer. The corrosion layer resistance, represented by R2, concerns the physical thickness of the corrosion layer obtained. Fig. 13 presents the EIS results in the form of Nyquist plots for the Pb1.5Sn0.05Ca and Pb1.5Sn0.12Bi samples after a cyclic voltammetry test corresponding to cycle 400, at a potential of +2.2V, for the purpose of shedding light on the conductivity behavior of the corrosion process and the layers formed. Table 2 shows the results obtained using the proposed equivalent circuit.

**Table 2:** EIS data for equivalent circuit.

Number of cycles 400 (+2.2V ESC)		
Parameter	Pb1.5Sn0.05Ca	Pb1.5Sn0.12Bi
R1 (Ω.cm^−2^)	1.692	1.664
Q1 CPE1 (F. cm^−2^)	0.03253	0.02682
CPE1(n1)	0.8878	0.8636
R2 (Ω.cm^−2^)	2.769	2.516
λ^2^	2.3x10^−3^	1.2x10^−3^

A potential of +2.2V corresponds to the charged state of the electrode. Under these conditions, PbO_2_ is formed, and this explains the low resistance values obtained. Fig. 13 shows the formation of capacitive arcs along with the arc obtained based on the equivalent circuit.

The two alloys presented very similar values for electrolyte resistance. This was to be expected, as they had both been prepared under the same conditions. The capacitive arc of the Pb1.5Sn0.05Ca alloy had a larger diameter than that of the Pb1.5Sn0.12Bi alloy, and this result may be attributed to the thicker layer of PbSO_4_ and PbO deposits, as demonstrated by cyclic voltammetry and gravimetric analysis. The layer of corrosion deposits forms a physical barrier for the electrolyte and causes an increase in the impedance of the system.

The constant phase element of the Pb1.5Sn0.05Ca alloy was also longer, and this may also be attributed to the development of a thicker corrosion layer that causes the electrical double layer.

The performance of the equivalent circuit provided a good approximation to the experimental results, confirming that the proposed circuit is capable of accurately describing the phenomenon that occurred, as outlined in studies by Burashnikova and colleagues [29].

**Fig. 14:**
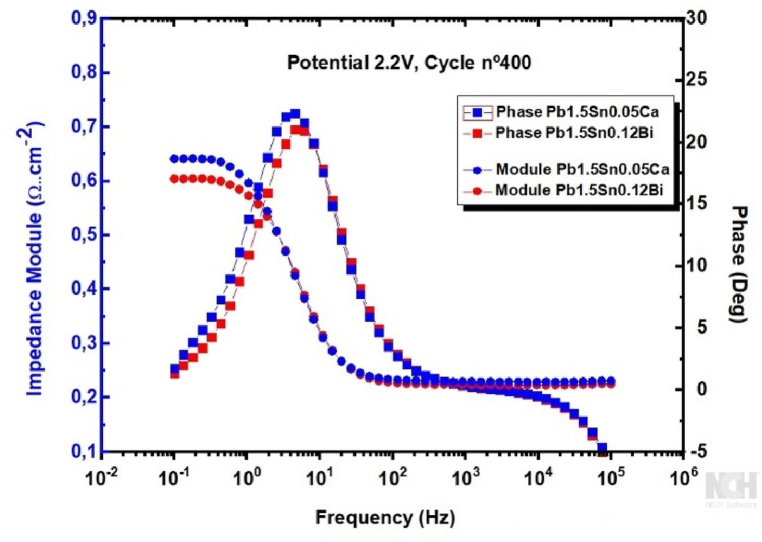
Bode-Modulus and Bode-Phase plots for Pb1.5Sn0.05Ca and Pb1.5Sn0.12Bi alloys with corrosion deposit films formed after cyclic voltammetry test (cycle 400).

Fig. 14 presents the EIS results in the form of a Bode plot, which shows the impedance modulus and phase angle as a function of frequency for the alloys under study, after a cyclic voltammetry test corresponding to cycle 400, at a potential of +2.2V, the purpose being to obtain more information about the corrosion layer of the materials.

In EIS studies of PbSn and PbSb alloys in an H_2_SO_4_ solution conducted by Osório et al. [27], the Bode phase plots showed two time constants: the first in the high-frequency zone, attributable to the reaction between the electrolyte and the phases rich in alloying elements (Sn or Sb), and a second associated with the reaction of the electrolyte with the lead-rich phases (matrix) [27,29].

In the frequency range of 10^2^–10^5^ Hz (medium to high frequencies), almost constant impedance modulus values are observed for both materials under study. The impedance values in the high-frequency region are attributable to electrolyte resistance, which remained close to 0.25 Ω cm^2^. In this frequency range, the phase angles observed were close to zero, indicating that the contribution provided by the phenomenon was purely resistive. This can be confirmed by the fact that phase angles equal to or close to 0° indicate the presence of resistors as circuit elements, while angles equal to or close to 90° indicate capacitive components [27,48]. The second constant was associated with alloy corrosion processes and changes in the passive layer formation observed at frequencies higher than 1Hz.

For frequencies of 10^2^ Hz and lower, a significant increase in the phase angle occurred, indicating the involvement of capacitive components in the system. This pronounced increase in the phase angle in the intermediate frequency region suggests modification of electrode surfaces and of the double electrical layer charging process, reaching a peak at frequencies close to 2 Hz [27,49].

Examination of the Bode-Module graph at lower frequencies revealed that the Pb1.5Sn0.12Bi alloy exhibited a lower impedance modulus compared to Pb1.5Sn0.05Ca. This result indicates that the deposit layer formed in the alloy has a more conductive nature. This is likely to be caused by the formation of Sn3Ca compounds, which decreases the extent to which tin oxides are formed. These oxides speed up conversion of PbSO_4_ to PbO_2_, thereby improving electrical mobility and enhancing battery recharge capacity.

The smaller maximum phase angle peak observed in the Pb1.5Sn0.12Bi alloy compared to that found for Pb1.5Sn0.05Ca may be associated with the greater compactness of the PbO_2_ layer, as shown above in Fig. 7 (c). A reduction in the porosity of the deposit layer facilitates the creation of a barrier to the electrolyte, thereby reducing the effects of charge accumulation between the pores, resulting in a decrease in the phase angle value.

## 4 Conclusions

These results lead us to conclude that calcium and bismuth act as grain refiners, with bismuth being more efficient. The Pb1.5Sn0.05Ca alloy exhibited more efficient mechanical properties so far as large-scale processing is concerned. This result can be attributed to the formation of Pb3Ca and Sn3Ca compounds. The Pb1.5Sn0.05Ca alloy presented superior mechanical properties in terms of both tensile strength and hardness.

The results of cyclic voltammetry indicated that both alloys display similar electrochemical phenomena, with the Pb1.5Sn0.05Ca alloy undergoing more extensive electrochemical corrosion and the Pb1.5Sn0.12Bi alloy greater oxygen evolution. Corrosion tended to occur at grain boundaries, and gravimetric testing corroborated the findings suggested by the voltametric peaks.

The SEM and XRD results indicated the formation of deposits at potentials of +1.3, +1.7, and +2.2V. The presence of PbSO_4_ crystals tended to be identified at a potential of +1.3V. At +1.7V, a smaller crystal size being observed in both tests. At +2.2V, significant morphological modification was apparent, with dissolution of the crystals formed, thereby confirming the deposition of a layer of PbO_2_.

The EIS tests confirmed the higher conductivity of the corrosion deposit in the PbSnBi alloy. This result can be attributed to a more compact corrosion and greater resistance in the formation of sulfate crystals at low potential. A chi-squared test showed that the equivalent circuit provided a good approximation. These results were consistent with those of the XRD and SEM tests. The Bode and Nyquist plots show that the corrosion of the PbSnCa alloy possesses greater electrical resistance than that of the PbSnBi alloy.

The Pb1.5Sn0.12Bi alloy exhibited better corrosion resistance properties compared to the Pb1.5Sn0.05Ca alloy. However, for its application, it would be necessary to use processing equipment with greater control over stresses, and the product can only be used in batteries that allow water replenishment.

Future work may investigate additives for electrolytes aimed at reducing water consumption or explore the application of the alloy in specialized lead battery applications. This is particularly relevant given the growing demand for vehicle electrification, energy storage systems, and integration between accumulators and renewable energy generation.

## CRediT authorship contribution statement

**Abdias Gomes dos Santos:** Writing – review & editing, Writing – original draft. **Magda Rosângela Santos Vieira:** Visualization, Validation, Supervision. **Flávio José da Silva:** Validation, Supervision, Methodology, Formal analysis. **Nadège Bouchonneau:** Visualization, Validation, Supervision, Project administration, Methodology, Investigation.

## Declaration of competing interest

The authors declare that they have no known competing financial interests or personal relationships that could have appeared to influence the work reported in this paper.
